# Continuous Performance Improvement Framework for Sustainable Wastewater Treatment Facilities in Arid Regions: Case of Wadi Rumah in Qassim, Saudi Arabia

**DOI:** 10.3390/ijerph18136857

**Published:** 2021-06-26

**Authors:** Husnain Haider, Mohammed AlHetari, Abdul Razzaq Ghumman, Ibrahim Saleh Al-Salamah, Hussein Thabit, Md. Shafiquzzaman

**Affiliations:** Department of Civil Engineering, College of Engineering, Qassim University, Buraydah 51452, Qassim, Saudi Arabia; malhitari83@gmail.com (M.A.); abdul.razzaq@qec.edu.sa (A.R.G.); alsalamah@qec.edu.sa (I.S.A.-S.); h.thabit@qu.edu.sa (H.T.); shafiq@qec.edu.sa (M.S.)

**Keywords:** arid regions, CCME-WQI, continuous performance improvement, grey rational analysis, performance benchmarking, wastewater treatment, water quality index

## Abstract

In arid regions such as Saudi Arabia, wastewater treatment (WWT) facilities (meeting promulgated standards) need to adapt their continuous performance improvement (CPI) for long-term sustainability. To achieve this, the facilities need to improve their performance to comply with more strict objectives for broader reuse applications of treated effluent. The present research proposes a CPI framework based on performance benchmarking process for the stepwise improvement of WWT facilities. A grey rational analysis water quality index (GWQI) based on exceedance probability was developed. For weights’ estimation of 11 physical, chemical, and biological water quality parameters, the entropy method effectively accommodated the changes in relative importance of the parameters with including additional future reuse applications. For existing effluent reuse scenarios of restricted and unrestricted irrigation, the GWQI values were found consistent with the modified version of the Canadian WQI (CWQI). The indices’ values (ranged between 0 and 100) greater than 80 showed the efficient operation of four WWT plants in the Qassim Region of Saudi Arabia. Two hypothetical CPI scenarios with future reuse applications (fish, livestock drinking, and recreation) showed an overall decline in the average (of four plants) values of the GWQI (97 to 78) and CWQI (85 to 60). CWQI predicted stricter results for the facilities with parameters’ concentrations exceeding the targets with larger margins and was found applicable for the CPI of WWT facilities in arid regions. For existing scenarios, the assessment results suggest the facilities to control and monitor the chlorination practice. For future targets, tertiary treatment needs to be enhanced for desired nutrients and total dissolved solids removal. The proposed CPI framework provides a platform to initiate the performance benchmarking process for WWT facilities at local or regional levels in Saudi Arabia and elsewhere.

## 1. Introduction

Surface water resources in the semi-arid and arid regions are facing challenges in meeting their intended uses, due to extreme low flows and the inadequate treatment of point and non-point pollution sources [1,2,3]. Depleting freshwater resources and low annual rainfalls have raised the need for wastewater reuse for different purposes (e.g., unrestricted and restricted irrigation, fishery, and recreation) based on the effluent quality produced by the wastewater treatment (WWT) plants [4,5]. To improve the circular economy and conserve water resources, the effluent standards are generally established based on the socio-economic benefits of wastewater reuse in a given geographical region [6,7]. In addition, wastewater reuse enhances agricultural production and minimizes the energy consumption that would otherwise be required for water production [6]. Climate change and population blast are enigmatically impacting available freshwater resources around the world. These impacts may be more catastrophic in semi-arid and arid regions, including Saudi Arabia. Overall, fresh surface water resources are limited in Saudi Arabia. Natural surface water channels (or rivers) are commonly called wadis in Arab and have been threatened by flash floods, long dry periods, and pollution from anthropogenic sources. Therefore, existing WWT facilities need to continuously improve their performances to comply with more stringent effluent standards for wider reuse applications in the future.

The main idea of continuous performance improvement (CPI) contains continuous and measurable improvement to achieve incremental progression through technology change or best practices [8]. The concept of CPI as a performance improvement tool was first introduced by Toyota in the 1950s [9]. CPI is suitable for (a) a process-oriented approach, i.e., improving individual processes, and (b) a result-oriented approach, i.e., improving the system based on final results. As most of the wastewater treatment plants (WWTPs) in the Saudi Arabia meet the national effluent standards (see results section), they are not experiencing any legislative or public pressure. Consequently, the managements of these facilities are not motivated or have no convincing reason for incremental improvement. However, a rational comparison of the effluent quality with more stringent standards for other reuse application can enhance their motivation toward CPI. A regional or national benchmarking process can be the catalyst for offering an incentive for CPI amongst the WWT facilities in the study area and the rest of the country.

The first step toward CPI is to evaluate the existing performance of the WWTPs. In treated wastewater, various physical, chemical, and biological water quality parameters (WQP), e.g., total suspended solids (TSS), biochemical oxygen demand (BOD), dissolved oxygen (DO) and coliforms, need to comply with the set effluent standards. The effluent standards are set for safe discharge to the natural environment and reuse of treated wastewater to protect the natural environment and human health [10,11,12]. Aggregated indices are commonly used around the world for water quality assessment, as they concisely inform of the health of natural systems and the performance of manmade systems [13,14,15]. Its simplicity of expressing the extensive water quality data into understandable terms (e.g., excellent, good, and poor) makes the information of measurements widely communicated. This is owing to the fact that, without requiring comprehensive knowledge about water science, WQI bridge the communication gap among scientists, decision makers, and society [16].

Different types of water quality indices were developed and used in the past to evaluate the performance of WWT facilities. In 2001, groups of Canadian Council of Ministers of Environment (CCME) developed a Canadian WQI, which is called the CWQI. The index was adapted from the original British Colombia WQI [17]. CWQI is flexible, and this appears by enabling the users from different countries to use site-specific standards and select them concerning WQP. The CWQI classifies water quality into five classes, i.e., excellent, good, fair, marginal, and poor. There are two primary limitations of the original CWQI. First, the formulation of CWQI does not consider parameter weight and uses an aggregation function based on three factors, namely, scope, frequency, and amplitude [18]. Second, it cannot include the bacteriological parameters, which are desired to be absent (nil or zero) in the sample. Furthermore, the concept of CPI is built on the idea of consistently improving the performance of a WWT facility to comply with more conservative water quality standards (WQS) in future. The relative importance of WQP potentially changes with a shift of the reuse application. For instance, bacteriological parameters are not highly important for restricted irrigation (for crops) but have to be absent for unrestricted irrigation (for raw vegetables consumable without cooking) or any other applications involving human contact (e.g., recreation and urban landscape irrigation) [19]. These conditions demand an assessment framework that can include bacteriological parameters in calculations as well as accommodate the changing relative importance (weights) of the WQP.

In the past, the CWQI was widely applied in the assessment of surface water [20], groundwater [21], water supply systems [14], and watersheds [22]. Gikas et al. [23] recently applied CWQI and the methodology of the Water Framework Directive proposed by the Ministry of Environment and Energy of Greece (WFD-MEEG) to assess the chemical status of a transboundary river. Their study found CCME-WQI to be more conservative (showed marginal to good water quality) than the WFD-MEEG, which resulted in a good class water quality. Similar results were reported by Zotou et al. [24] for a river water quality assessment in Greece, where CWQI was found to be relatively stricter out of seven indices. Hansda et al. [25] applied CWQI to the Khadakwasla Reservoir to identify water quality trends, using physicochemical parameters. Lumb et al. [20] compared two U.S.-based indices (additive and multiplicative) and Oregon WQI (harmonic averaging) with CWQI for the water quality assessment of 30 river locations in Ontario, Canada. They found close results for Oregon WQI and CWQI, which were overall stricter than the U.S.-based indices. Overall, the literature reports scant studies on the assessment of WWT facilities. Jamshidzadeh and Barzi [26] developed three WQI for assessing the suitability of treated effluent for agricultural suitability in Iran. The three indices, viz., overall WQI, acceptability WQI, and health WQI, based on different weighted aggregation functions, were applied for spatiotemporal water quality evaluation. Original CWQI and multivariate statistical analysis were used to assess the suitability of treated effluents for irrigation use [27,28,29].

The concept of CPI integrated with CWQI was first introduced by Bereskie et al. [8] for small water supply systems in Canada operating with minimal treatment, due to operational and financial limitations. They projected the water quality improvements, in terms of CWQI, with increasing source water protection, level of treatment, and distribution system management. The CPI concept has not been applied to the WWT facilities in arid regions, which need consistent improvements for conforming with stricter reuse standards for wider applications in the future.

The Kingdom of Saudi Arabia (KSA) is a well-known country of the Arabian Gulf: it is located in an arid region, has water shortage and limited groundwater resources [30,31]. Surface water availability is limited in the country, owed to low average annual rainfall, ranging between 50 mm and 150 mm, and high evapotranspiration rates varying from 3000 to more than 4200 mm/year [32,33]. KSA is experiencing high population growth rates and going through rapid urbanization, industrial, and agricultural developments that are constantly raising water demands. To meet these ever-growing demands, the limited water resources available in the country are under continuous water stress. Around 87% of the total extracted water (from desalination and surface and groundwater) is being used to meet the agricultural needs of the country [34]. Faced with the most water consumption being contributed to the agriculture sector, the valuable freshwater resources are facing intense depletion in the country.

Such continuous groundwater depletion implicates the future direction to more expenditures on desalinated water to satisfy the growing demands. The government of Saudi Arabia aimed to reuse 100% of treated wastewater from cities with a population equal to or higher than 5000 by 2025 [35]. Thus, effective reuse of treated effluents from local WWT facilities is essential for the country to confront the water shortage instead of disposing them into wadis and water bodies. Presently, the primary use of treated wastewater is agricultural and landscaping applications. As per the General Electric Industry’s white paper on water scarcity in Saudi Arabia, the treated wastewater reuse is approximately 2367 million m^3^/day, which represents 40% of municipal wastewater [36]. With regard to the efficiency of WWT facilities in KSA, limited studies explored the compliance of treated effluents for reuse. Shawky and Sbiany [37] assessed the suitability of the Al-Khobar wastewater treatment plant’s effluent for agriculture. Before effluent reuse, the compliance of the treated effluent needs to be continuously monitored against the established local standards. The application of CPI-based benchmarking of WWT facilities provide a rationale for upgrading the treatment levels, which will enhance the sustainability of water resources in arid regions.

We did not find any application of CPI of wastewater treatment facilities in Saudi Arabia. The original CWQI first evaluates the scope (i.e., percentage of failed variables), frequency (i.e., percentage of failed tests), and amplitude (i.e., how much the failed tests’ values are away from the objective value) and aggregates all this information into an index. The CPI concept is based on pushing the effluent standards toward more stringent targets for wider reuse applications. The importance of parameters might change with improved targets, for instance, the significance of microbiological parameters for unrestricted and restricted irrigation is certainly different. For all the parameters of concern in long monitoring data, the exceedance probability (*P_e_*) of a water quality failure provides an insight into the probability of exceeding the standard value. In the present study, we develop various scenarios for effluent reuse, estimate *P_e_* for all these scenarios, and convert *P_e_* into non-exceedance probabilities (*P*) (i.e., benefit criteria). Being adaptable for CPI application, the entropy method estimates the weights of the WQPs for each scenario. Subsequently, the grey rational analysis (GRA) aggregates the *P* for all the parameters; the index is named the GRA water quality index (GWQI). The study also compares the modified CWQI and GWQI for each scenario and identified the improvement actions for the CPI of wastewater treatment facilities along Wadi Rumah in Qassim Region of Saudi Arabia and other arid regions.

## 2. Materials and Methods

### 2.1. Continuous Performance Improvement Framework

**Figure 1:**
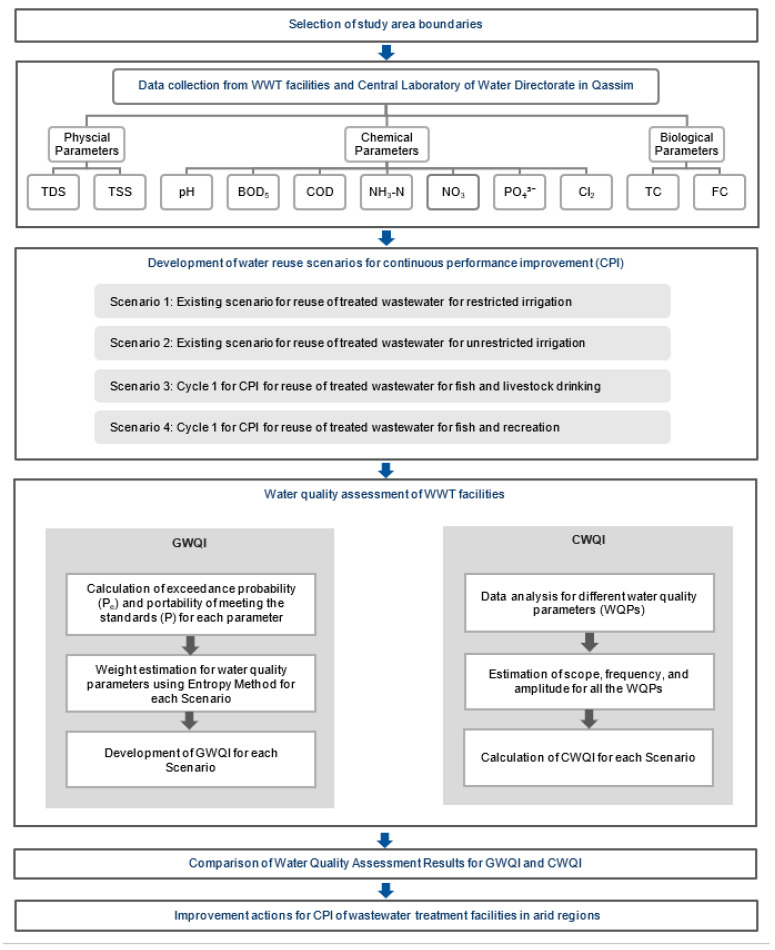
Continuous performance improvement framework for wastewater treatment facilities in arid regions.

The CPI framework developed in this study is described in Figure 1. The framework initiates with defining the study area’s boundaries and the selection of WWT facilities. Effluent water quality data for various physical, chemical, and biological WQPs were obtained from the selected WWT facilities and the Central Laboratory of the Water Directorate of the study area. Different scenarios for reuse applications of the treated effluents were developed for CPI of the WWT facilities. For each scenario, two water quality indices, namely, GWQI and CWQI, were calculated to evaluate the performance of the WWT facilities. For CPI, lacking WQPs were identified, and improvement actions are suggested for each treatment facility.

### 2.2. Study Area

Wadi Rumah is a seasonal stream in Saudi Arabia that collects surface runoff from urban and agricultural watersheds. Approximately 2000 km long, Wadi Rumah is one of longest wadis in Saudi Arabia. A long stretch of the wadi passes through the Qassim Region that has its own significance, due to extensive agricultural activities. Being in the heart of the country, livestock and farms further enhance the value of the region. Primarily during the winter (rainy) season, the wadi provides additional beneficial uses, including domestic, fish, recreation, and natural groundwater recharge. To avoid the negative impacts of wastewater discharge on these beneficial uses, several WWT facilities are installed along the entire length of the wadi.

**Figure 2:**
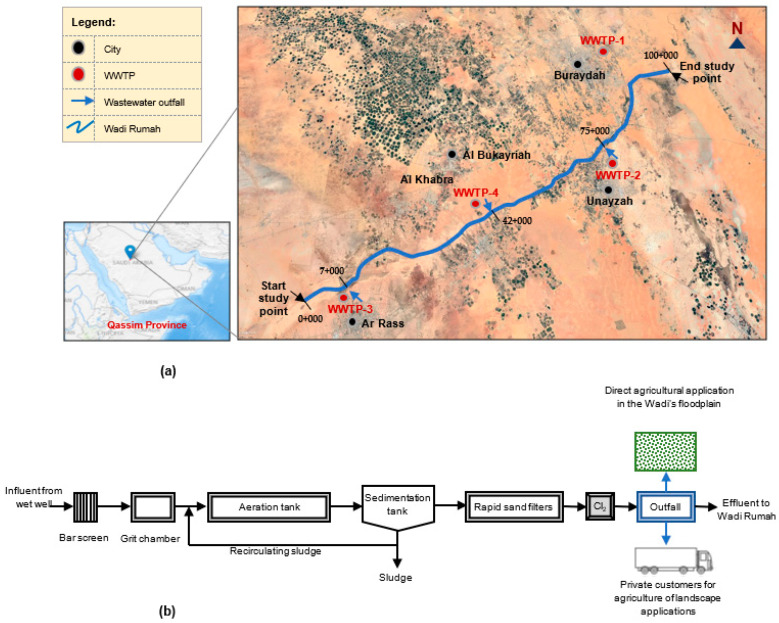
Study area. (**a**) Map showing location of four wastewater treatment plants discharging in Wadi Rumah (not to scale), (**b**) a typical wastewater treatment process diagram of the facilities in study area.

Figure 2a shows the boundaries of the study area that includes four wastewater treatment plants (WWTPs). WWTP1 and WWTP2 are located north-east of the cities of Buraydah and Unayzah, respectively. WWTP3 is located north-west of Al Rass city. WWTP4, located south-east of Al Bukayriah city, serves the population of Al Bukayriah, Al Khabra, and Riyadh Al Khabra governorates. In the existing scenario, most of the treated effluent is used for agricultural and landscaping applications. In most cases, the customers directly fill the containers of their trucks at the WWT outfall. The direct discharge of the outfall overflows into the wadi and forms a condition similar to a pond during the dry season (summer), while the overflows mix with wadi water during the wet season (winter). Figure 2b illustrates a typical process flow diagram of the four WWTPs operating in the study area. The average tertiary treated effluents during the study period for WWTP-1, WWTP-2, WWTP-3, and WWTP-4 are 140,347, 35,212, 24,795, and 10,408 m^3^/day respectively. Figure 2a shows approximate positions of the four WWTPs located along 100 km length (shown with thick blue line) of the wadi. Preliminary and primary treatment units are the bar screen and grit chamber, followed by an extended aeration type activated sludge process for secondary treatment. Finally, the tertiary level treatment consists of rapid sand filters for polishing and chlorine disinfection.

### 2.3. Development of Scenarios for CPI

**Figure 3:**
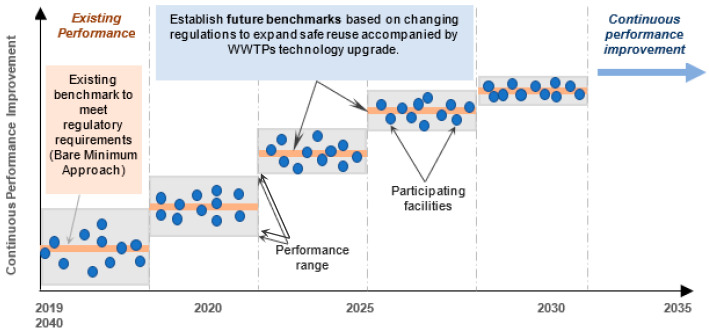
The concept illustrating continuous performance improvement for wastewater treatment facilities in arid regions (modified after [8]).

Describing the concept of CPI in Figure 3, the selected WWTPs are assumed to be participating facilities and their corresponding performance is represented by the blue dots. The solid line represents the average index value, which is essentially a benchmark for a given assessment period, e.g., 2019–2020. The existing benchmark indicates the current status of treatment for WWTPs that represents the average performance of the WWT facilities controlled by a set of regulatory standards for designated reuse application. The existing effluents’ concentrations were compared with more stringent WQS for sustainable wastewater reuse. This comparison potentially works as a stimulus for the performance improvement of WWTPs to meet the desired standards for additional reuse applications in future benchmarking cycles. The facilities performing lower than benchmarks need major improvements to match with better-performing facilities as well as more stringent future standards. The facilities performing higher than the benchmark may need moderate improvements to meet the established standards for the upcoming cycles. This exercise also brings all the participating facilities closer to the benchmark.

Table 1 defines different scenarios for existing and future improvements of treated effluents reuse. The scenarios in Table 1 clearly show incremental additions in the reuse applications for future benchmarking cycles. The scenarios were established based on the potential beneficial uses in the region. The first two scenarios (S1 and S2) denote the existing applicable standards for restricted and unrestricted irrigation in Saudi Arabia, while the other two scenarios (S3 and S4) are defined for CPI. The performance improvement could be implemented within a hypothetically specified 5-year time interval of the scenario improvement cycle, depending on the assessment results for future reuse applications. The nature of applied improvement depends on the target as to what contaminant should be removed and the corresponding specific treatment processes required within the WWT facilities.

### 2.4. Water Quality Parameters, Standards, and Analytical Methods

One-year treated effluent data for selected physical, chemical, and biological WQP were obtained for the assessment of WWTPs. The physical water quality data include total dissolved solids (TDS) and total suspended solids (TSS). Chemical parameters are pH, biological oxygen demand (BOD_5_), chemical oxygen demand (COD), ammonia nitrogen (NH_3_–N), nitrate nitrogen (NO_3_–N), phosphates (PO_4_–), and residual chlorine (Cl_2_). The biological parameters include total coliforms (TC) and fecal coliforms (FC). These eleven parameters of the WWTPs are routinely monitored by the facilities and the Central Laboratory of the Water Directorate in Qassim Region. The parameters are well known for their impacts on the environment and human health. Table 2 shows the standards (or guideline values) for these parameters for the various scenarios defined in Table 1.

The physical parameters have impacts with varying significance, according to the reuse applications, particularly for scenario 1 and scenario 2. The quality of the irrigated water and soil are the main media to support the growth and yield of the plants. Plant growth can be affected indirectly by the quality of treated effluents. For example, the presence of high TDS in treated effluents may increase soil salinity at the disposal sites [47]. The salinity can accumulate at plant roots and cause osmotic effects, thus reducing plant nutrient uptake and consequently its growth [48]. Additionally, with long-term application of wastewater, TSS and other organics can change the soil properties [49]. For scenario 3, the TDS levels less than 1000 mg/l have no serious burden to livestock or poultry [50].

The chemical parameters impact the soil and plants more considerably in scenarios 2, 3, and 4. The disposal of inadequately treated effluents can modify the soil characteristics. For example, effluent organic matter indicators (e.g., BOD_5_ and COD) can alter soil properties [49]. Although there are benefits of sewage effluent nutrients (i.e., nitrogen and phosphorus) to plant yields, excessive nutrients can block the soil pores and enhance the eutrophication process in water bodies [51,52]. Moreover, treated effluents can sometimes increase organic matter and decrease pH and the infiltration rate at disposal sites [37,52]. Regarding scenario 3 of livestock drinking, EPA Victoria guidelines (Australia) prevent blue-green algae blooms in stored, reclaimed water [53,54,55]. Blue-green algae develops in stagnant water because of the presence of nutrients (upper limit for NO_2_–N plus NO_3_–N is 100 mg/L), which can cause diseases and death of livestock [49]. The pollutants regarding scenario 4 of recreation activities and fishery class are adopted from some WQS of Malaysia for ammonia, BOD, and COD concentrations for CPI of WWTPs.

Public health is the greatest concern that needs to be addressed carefully in all treated effluent reuse applications [56]. The presence of improperly controlled microorganisms and pollutants can have a wide range of health risks through direct contact with wastewater effluents or indirect contact via ingestion of contaminated food or crop products [57]. The biological WQP, such as TC and FC, must be efficiently removed because of their cycles of infection among plants, livestock, and the public, particularly for scenarios 2, 3, and 4. Pathogens from secondary treated effluents may pose negligible health concerns to the soil, especially in arid and semi-arid regions [58]. In arid regions, such as the study area, wastewater effluents are discharged into wadi channels, forming ponds during dry periods. The locals living near these areas and farm workers or WWTPs workers who have direct contact with treated wastewater effluents can be affected by the presence of pathogens. Moreover, the effluents ponding water in the dry channel during dry periods can be accessed by wild animals and grazing livestock. The livestock can be affected by the quality of treated wastewater effluents and pose adverse effects to the public when consuming their milk or meat. Poppenga et al. [59] found insufficient evidence to establish that the reuse of disinfected tertiary treatment for livestock watering poses a significant risk to public health. Consequently, more stringent WQS are proposed in Table 2 for scenarios 3 and 4.

All parameters were tested as per the standard methods for the examination of water and wastewater analysis prescribed by American Public Health Association [60]. The HACH 440d multi-parameter meter was used to measure TDS and pH [14]. TSS was determined by the gravimetric method. The HACH-DR 5000^TM^ UV–Vis spectrophotometer (USA) was used to measure NH_3_–N (direct ISE method), NO_3_–N, COD (reactor digestion method), PO_4_–P (acid persulfate digestion method), and TSS (photometric method) [61]. Fecal coliform (FC) and total coliform (TC) were measured, using a Quanti-Tray, which consists of 51 individually sealable cells. BOD5 analyses were performed as per the APHA standard dilution method [60].

### 2.5. Grey Rational Analysis-Water Quality Index (GWQI)

The grey system theory was firstly introduced by Deng in 1982 to deal with a system that has known and unknown information [62]. Grey relational analysis (GRA) is the extension of grey system theory and is used as an assessment tool in different fields, including economics and engineering. The present research integrates the entropy method with GRA to develop GWQI for the performance assessment of WWT facilities.

The following steps were adopted for the development of GWQI:

Step 1: Calculate the probability of meeting the desired standards for WWT plants’ effluents.

First, estimate the exceedance probability (*P_e_*) of water quality failure for each parameter for a given scenario (reuse application), using the following equation:

(1) $$P_{e}=\frac{m}{n+1}$$

where ‘*m*’ is the rank of the measured parameter after sorting the data set and ‘*n’* is the number of times that a parameter was monitored.

Now, find the non-exceedance probability (*P*) for each parameter using the following equation:

(2) $$P=1-P_{e}$$

Step 2: Estimation of weights using entropy methods.

The present research demanded a unique sequence of weights for the selected WQP. Therefore, the entropy method [63] was used to estimate the importance weights for the parameters in Table 2 for the wastewater reuse scenarios described in Table 1. Each parameter was scored, using the 10-points Likert scale given in Table 3, against four evaluation criteria, including C1—impact on geoenvironment, C2—impact on plant growth, C3—impact on livestock safety, and C4—impact on possible human contact. The criteria were scored by the water engineering and management experts from the profession and academia.

If the evaluation criteria for treated effluents impacts are ‘*j*’ for the parameter ‘*i*’, a scoring matrix can be generated for the importance of all the parameters relevant to its effluent reuse scenario. The step-by-step procedure for weight estimation is presented in the following.

Step 2.1: Develop the entropy matrix (*Ej*)

The matrix can be generated using the following equation:

(3) $$E_{j}=-\alpha \sum_{i=1}^{n}P_{ij}ln\left(P_{ij}\right)\;\mathrm{for}\;\mathrm{all}\;j$$

where ‘*α*’ is a constant for ‘*n*’. ‘*n*’ here is the number of evaluation criteria which confirms that all entropies ‘*E_j_*’ varies between ‘0’ and ‘1’. The constant ‘*α*’ is defined as follows:

(4) $$\alpha =\frac{1}{\ln\left(n\right)}$$

Step 2.2: Estimate the importance weights and degree of diversity.

The importance weights for all parameters were estimated using the following equation:

(5) $$w_{j}=d_{j}/\sum_{i=1}^{k}d_{j};\;\forall \;j$$

where ‘*d_j_*’ is the degree of diversity amongst the water experts in a given evaluation criterion ‘*j*’, and is defined as follows:

(6) $$d_{j}=1-E_{j}$$

Step 2.3: Estimate the final weights.

For each scenario, a priori for all the parameters was allocated from the water experts for the subjective weights ${\hat{w}}_{j}$ defined in Table 3.

The final weight for a parameter is relevant to its importance for different effluent reuse scenarios and was estimated using the following equation:

(7) $$W_{j}=\frac{{\hat{w}}_{j}w_{j}}{\sum_{i=1}^{k}{\hat{w}}_{j}w_{j}}$$

Sum of the weights for all parameters should be equal to 1.

Step 3: Perform grey rational analysis (GRA) reference.

In the present research, each WWT facility’s performance was analyzed using the GRA approach given by Cenglin [64]. The steps are given in the following.

Step 3.1: Determination of grey relational coefficient.

Determination of grey relational coefficient for all selected WQP of each WWTP relative to reference point (*P* = 100) was carried out, using the following equation proposed by Deng [65].

(8) $$\xi_{i}\left(k\right)=\frac{\underset{i}{\min}\;\underset{k}{\min}\left|x_{0}\left(k\right)-x_{i}\left(k\right)\right|+\xi \;\underset{i}{\max}\;\underset{k}{\max}\left|x_{0}\left(k\right)-x_{i}\left(k\right)\right|}{\left|x_{0}\left(k\right)-x_{i}\left(k\right)\right|+\xi \underset{i}{\max}\;\underset{k}{\max}\left|x_{0}\left(k\right)-x_{i}\left(k\right)\right|}$$

where $\xi_{i}\left(k\right)$ is the grey relational coefficient of *x_i_* to *x*_0_ in *k* moments, and represents the relative difference of the comparative sequence *x_i_* and reference sequence *x*_o_ in ‘*k*’ moments for a parameter *i*. The *ξ* is called the distinguish coefficient and in *ξ* ϵ [0,1] and in this study it is taken as *ξ* = 0.5 to offer moderate effects.

Step 3.2: Compute the grey relational grade.

The final grey relational grade that represents here the GWQI was computed by adding the product of each parameter entropy weight (estimated in Step 2.3) by its grey relational coefficient as follows:

(9) $$GWQI=\sum W_{j}\times \xi_{i}\left(k\right)$$

### 2.6. Canadian Council of Ministers of Environment Water Quality Index (CWQI)

CWQI was selected to assess the quality of effluents of wastewater treatment facilities because it has flexible application to many water resources and is capable of adaptation with different WQS.

The calculation of CWQI is obtained as Equation (10) [18].

(10) $$CWQI=100-\left(\frac{\sqrt{F_{1}^{2}+F_{2}^{2}+F_{3}^{2}}}{1.732}\right)$$

The divisor of 1.732 normalizes the scale of the index to a range between 0 and 100 and was introduced to prevent the resultant scale of the index from reaching a maximum of 173.2 because each factor can reach a value of 100.

The CWQI calculation is based on a combination of *F*_1_, *F*_2_, and *F*_3_ factors.

*F*_1_ (Scope): Describes the level of non-compliance of all the WQP in a given assessment period and can be estimated using the following equation:

(11) $$F_{1}=\left(\frac{Number\;of\;failed\;variables}{Total\;number\;of\;variables}\right)\times 100$$

where the number of failed variables is essentially the WQP that exceeded their target values (objectives).

*F*_2_ (Frequency): Estimates the percentage of failed tests, i.e., individual tests for all the WQP that did not meet the target or objective values. *F*_2_ can be calculated as follows:

(12) $$F_{2}=\left(\frac{Number\;of\;failed\;tests}{Total\;number\;of\;tests}\right)\times 100$$

*F*_3_ (Amplitude): Represents the amount by which the failed test values did not meet the target value. *F*_3_ is an asymptotic function that scales the *nse* between 0 and 100, so that *F*_3_ can be analogous to *F*_1_ and *F*_2_*. F*_3_ is calculated using the following equation:

(13) $$F_{3}=\left(\frac{nse}{0.01\;nse+0.01}\right)$$

Calculation of *F*_3_ is a two-step process:

Step 1: Calculate excursion, which represents the number of times an individual water quality parameter was found to be greater than (or less than) the objective. In the case that water quality parameters are desired to be less than the objective, the excursion is estimated as follows:

(14) $$excursion_{i}=\left(\frac{Failed\;test\;value_{i}}{Objective_{i}}\right)-1$$

In the case that water quality parameters are desired to be higher than the objective, e.g., residual chlorine, the excursion is estimated as follows:

(15) $$excursion_{i}=\left(\frac{Objective_{i}}{Failed\;test\;value_{i}}\right)-1$$

Step 2: Calculate normalized sum of excursion (*nse*) using the following equation:

(16) $$nse=\frac{\sum_{i=1}^{n}excursion_{i}}{\#\;of\;tests}$$

Finally, via substitution of the above equations into Equation (10), CWQI was obtained for each WWT facility (for each scenario) and categorized as described in Table 4.

As Equation (10) cannot adapt a complete absence (target value) of microbiological parameters, the following modification to the original CWQI is proposed [14]:

(17) $${\left(WQI\right)}_{MB}=\left[1-\left(\frac{Total\;number\;of\;failed\;microbiological\;tests}{Total\;number\;of\;microbiological\;tests}\right)\right]\times 100$$

where *WQI_MB_* is the microbiological water quality index.

Step 5: Finally, the modified CWQI can be calculated as follows:

(18) $$Modified\;CWQI=W_{1}\times CWQI_{PC}+W_{2}\times WQI_{MB}$$

where *W*_1_ and *W*_2_ are the relative importance weights for *CWQI_PC_* and *WQI_MB_*. Table 5 presents the weighting scheme for the modified CWQI based on the relative importance of *WQI_MB_* for each scenario for CPI. Finally, the modified GWQI results are categorized as per Table 2 and compared with GWQI to facilitate the participating facilities for CPI. Decision makers can take effective improvement actions, based on the benchmarking results. Please note that CWQI in the following text represents the modified CWQI.

## 3. Results

### 3.1. Water Quality Monitoring

Table 6 presents the summary of water quality monitoring data for the four WWT facilities located within the study area boundary as defined in Figure 2. Minimum (MIN), mean (MEAN), maximum (MAX), standard deviation (SD), and the coefficient of variation (CV) for all the WQPs are presented in Table 6. The average TSS in all the plants’ effluents meet the most stringent reuse standards for scenario 4 (i.e., 5 mg/L for recreation use) with occasional slightly higher values in the case of WWT-3 and WWTP-4. Presently, average TDS levels meet the target concentration of 1500 mg/L, which fulfills the requirements for first three scenarios. Maximum TDS levels in WWTP-2 and WWTP-3 comply with the target concentrations for scenario 1 and scenario 2, while the effluent of WWTP-4 meets the target concentration for scenario 3 as well. Occasionally, the WWTP-1 was found to be noncompliant with all the scenarios with the highest value of 3263 mg/L.

In most cases, the pH values lie within the desired range for all the four scenarios with occasionally higher values than 8, i.e., the maximum allowable target value for scenario 4. Average BOD values for WWTP-1 and WWTP-2 meet the target values for all four scenarios, while the remaining two facilities comply with the targets for the first three scenarios. However, the maximum observed concentrations are higher than the target concentration of 5 mg/L for fish and livestock drinking (S3). Likewise, the COD values (both average and maximum) are lower than the target concentration of 25 mg/L for scenario 4 in the effluents of WWTP-1 and WWTP-2. Although the average COD values for WWTP-3 and WWTP-4 are lower than 25 mg/L (S4), the maximum values are slightly higher than the target value. Average NH_3_–N concentration in the effluents of all the facilities are less than the target level of 0.3 mg/L for scenario 4, except WWTP-1, which shows a slightly higher value, i.e., 0.5 mg/L. The effluents of all the facilities meet the targets for all the scenarios in terms of average NO_3_ concentration. In terms of PO_4_ levels, the average values comply with the existing reuse standards for scenario 1 and scenario 2. The residual chlorine values are found higher than the desired range at all the facilities. Nevertheless, high chlorine always yields the absence of TC and FC in treated effluents.

### 3.2. Water Quality Indices

Following the methodology described in Section 2.3, Table 7 presents the calculated non-exceedance probabilities (*P*) of all the WQPs for all the WWT facilities. For the first two (existing) scenarios, the results presented in Table 7 show overall high *P* values for all the WQPs, except for residual chlorine. Phosphate failures were observed for S3 and S4 while BOD, NH_3_–N, and NO_3_–N also were found to be higher than the scenario 4 target values. Table 8 shows the importance weights of the WQPs for each scenario, using the entropy method. An example of weights estimation for scenario 1 (restricted irrigation) is illustrated in Appendix A. For the first two scenarios, more weights were assigned to physical and chemical WQPs, while a more balanced weights distribution was adopted for S3 and S4. Finally, GWQI was calculated for all the participating facilities using Equation (9).

**Figure 4:**
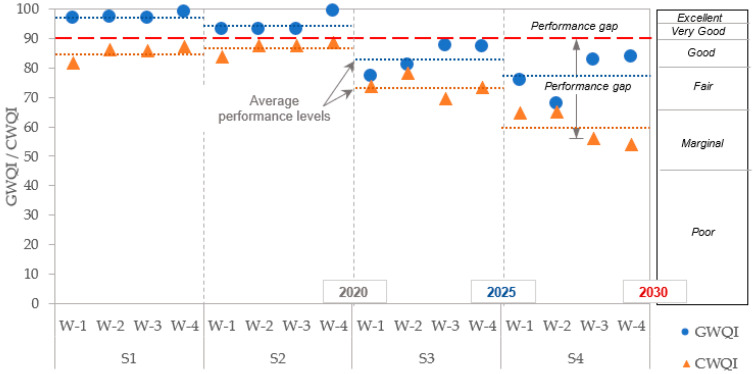
GWQI, CWQI, and identification of performance gap for CPI. S—Existing scenario for reuse of treated wastewater for restricted irrigation, S2—Existing scenario for reuse of treated wastewater for unrestricted irrigation, S3—Cycle 1 for CPI for reuse of treated wastewater for fish and livestock drinking, S4—Cycle 1 for CPI for reuse of treated wastewater for fish and recreation. Note: “W” in the figure denotes wastewater treatment plant.

Equations (11)–(13) calculated the scope (F1), frequency (F2) and amplitude (F3) for the given data. Subsequently, Equation (10) estimated the CWQI_PC_ and Equation (11) calculated the WQI_MB_ for all the facilities. Finally, CWQI was calculated for all the facilities using Equation (10). Figure 4 illustrates the calculated GWQI (circular markers) and CWQI (triangular markers) for all the four scenarios. It can be seen in the figure that GWQI shows overall higher values than the CWQI for all the treatment facilities. These finding are similar to a recent study of Gikas et al. [23], who measured almost the same WQPs used in the present study in a large transboundary river, using the Ministry of Environment and Energy of Greece (WFD-MEEG) and CWQI. As the investigations found CWQI to be a stricter WQI, they recommend the use of CWQI for river water quality control.

### 3.3. Continuous Performance Improvement

Figure 4 presents the calculated GWQI and CWQI for the four CPI scenarios. Based on the description given in Table 4, the benchmark can be presumed at ‘90’ for ‘very high’ or ‘excellent’ performance levels. Moving toward CPI with existing WWT practices, a continuous performance decline can be observed for S3 and S4. It is noteworthy that slight improvement in CWQI for S2 attributes to the distribution of importance weights of WQI_MB_ (increase from 0.1 for S1, to 0.2 for S2) and the complete absence of TC and FC. It is interesting to note that WWTP-3 and WWTP-4 showed higher CWQI values for S1 and S2, while their performance declined to comply with the more stringent effluent standards in the case of S3 and S4. For GWQI, all the facilities performed higher than the benchmark for S1 and S2, while the performance gap (difference between the index value and the benchmark, also see Figure 4 for WWTP-1 and WWTP-2) is higher than the other two facilities. Moreover, both the indices (GWQI and CWQI) are close for WWTP-1 and WWTP-2. For WWTP-3 and WWTP-4, amplitude (F3) in CWQI primarily caused the difference between the two indices. Amplitude includes “excursion” in its formulation that essentially assesses the amount at which the measured value is higher than the target concentration. The GWQI formulation is based on the exceedance probability that mainly captures the frequency (F2) function of CWQI. However, the flexibility of assigning the importance weights to the WQPs is an advantage of GWQI over CWQI. The weights assignment becomes important with the inclusion of additional reuse applications in future scenarios for CPI.

The WWT facilities performing higher than the benchmark but laying in the “very good” range primarily need to maintain their performance with minor improvements, e.g., monitoring frequency and proactive maintenance practices, which can further minimize the number of failure incidents and lift the performance to “excellent” zone. The facilities in the “excellent” performance zone primarily need to maintain their performance. The facilities performing lower than the benchmark need to adopt the recommended actions given in Table 4 correspond to their performance level.

## 4. Discussion

The improvement of wastewater treatment and the safe applicability of treated wastewater has attracted more attention recently and is linked to circular economy and socio-economic development. Locally in KSA, lack of knowledge on the performance of WWTPs in removing pollutants has produced reluctance to reuse treated wastewater in public. This needs the implementing of strategic management and changing public awareness of the negative perception about reusing treated wastewater [66]. The Saudi government has set wastewater reuse or discharge standards to protect human health and the surrounding environment. For example, KSA has set standards for the reuse of wastewater in different fields, such as restricted and unrestricted irrigation. Lack of water supply resources for the irrigation of crops impacts agricultural output [32]. As such, treated wastewater reuse is necessary, especially for water-stressed countries that rely on groundwater and costly produced desalinated water. Currently, reevaluation of wastewater reuse and reducing energy consumed by desalination plants is urgent for effective water management in KSA [67].

Prior to reuse, the compliance of treatment plant effluents for agricultural irrigation needs to be checked since it could be violated [51]. For instance, microbial risk can be found, even after the chlorination process for a conventional wastewater treatment process and consequently, makes it not suitable for unrestricted irrigation [68]. Environmental impacts, such as plant cover as a bio-indicator of pollution and soil deterioration is another challenging problem for WWTPs [38]. These reuse concerns related to the public, environment, and economy should be overcome to benefit from treated effluents. Thus, monitoring the current status of WWTPs treated effluents based on local designated standards of reuse applications associated with CPI in the future based on performance benchmarking of more stringent international and organizational WQS for higher reuse is an important requirement.

The results presented in Figure 4 clearly shows that the four participating WWT facilities in the Qassim region sufficiently (*P* > 95% in Table 7) meet the existing reuse standards of restricted irrigation (S1) and unrestricted irrigation (S2). Average performance levels (see Figure 4) for GWQI are higher than the presumed benchmark (i.e., 90), while it is slightly lower in the case of CWQI. The facilities primarily need to focus on controlling the chlorine dose that effectively removes the biological contaminants (TC and FC), but higher concentrations are not suitable for plants and aquatic life. It is worth mentioning that during the field visits to the WWT plants, we observed fish in the ponds of treated effluents. The type of fish was *Nile tilapia Oreochromis niloticus*, thus the ammonia toxicity to this kind of fish should be taken care of with compliance to the chronic toxicity range taken from the experimental study [69]. Moreover, the range of residual chlorine levels are higher than the objective values and could be toxic to fish life. Increasing, the chlorine contact time in treated effluent storage can resolve this problem.

The objective of CPI is to improve or upgrade the performance to comply with more stringent effluent standards for wider reuse applications, such as fish, livestock drinking, and recreation. Table 7 provides important information about the facilities’ performance in terms of water quality compliance and noncompliance for all scenarios. Overall, for S3, the main WQPs that were found to be noncompliant (in addition to residual Cl_2_) with the treated effluents’ objectives are BOD for WWTP-3 and WWTP-4 and PO_4_ for all the facilities. BOD values were found to be slightly higher than the objective value of 5 mg/L in some cases; maintaining the process parameters through appropriate monitoring can help in achieving the target. For extended aeration type activated sludge process, the pH, temperature, DO, and return sludge are the most important process parameters to be controlled [70]. Although higher concentrations of PO_4_ (10 mg/L for S1 and S2) are suitable for irrigation applications, these levels enhance the eutrophication process in the wastewater ponds. Eutrophication impedes the reaeration process, resulting in a water quality not suitable for fish survival. More water quality violations in the case of scenario 4 suggest interventions or upgradations for CPI. For instance, all the facilities were found to be noncompliant with the S4 objective for TDS. Presently, facilities are operating with sand filtration as a tertiary level treatment process, which essentially is the pretreatment for reverse osmosis (RO) processes. RO can be added in future to meet S4 targets for Cycle 2 of CPI. In addition to TDS removal, RO can effectively remove dissolved organics for secondary treated effluent and used for the effluent polishing [71]. Table 9 presents the recommended improvement actions for CPI of WWT facilities in arid regions. As CWQI was found stricter than the GWQI, the improvement actions are recommended based on the CWQI results.

The CPI framework helps facilities’ management in the compliance assessment of WWTPs. This exercise identifies the gap between the existing performance and the desired treated effluents’ objectives for various reuse applications in arid regions. Land use changes can alter the hydrological cycle and subsequently disrupt the spatiotemporal flows in surface waters [5]. The CPI process should consider this phenomenon, as extremely low wadi flows demand more strict discharge regulations. In addition to arid regions, the proposed framework can be adopted in other parts of the world facing water quality problems, e.g., the Southwest U.S. and Small Islands Developing States (SIDS). For instance, the freshwater resources in SIDS are seriously threatened by climate change. Being surrounded by the ocean, more than 70% of SIDS suffer water shortages and groundwater pollution problems [72]. Kang et al. [73] reported the presence of high TDS levels (1000 to 10,000 mg/L) in the deep groundwater resources of 7 out of 17 basins in the Great Basin of the Southwest U.S. In such regions, the expansive treatment of brackish (or saline) water urges for increasing the wastewater reuse for socio-economic and environmental sustainability.

## 5. Conclusions

The WWT facilities in arid regions need to adapt their continuous performance improvement framework for wider applications of treated effluents. The water quality index based on exceedance probability (the effluent concentration exceeding the objective concentration) was developed using grey rational analysis and named GWQI. For estimation of WQPs’ weights, the entropy method was found suitable for CPI application, as the relative importance of the parameters changes with including additional reuse applications in the future. For existing effluent reuse scenarios, the GWQI assessment results (for two physical, seven chemical, and two biological WQPs) were found to be in agreement with the modified version of the well-known CWQI. Higher than 80 values of both the GWQI and CWQI showed that WWTPs in the Qassim Region of Saudi Arabia are effectively meeting the existing promulgated standards for restricted irrigation and unrestricted irrigation. These findings show that the facilities are effectively managing the treatment processes to control the objective effluent concentrations.

For evaluating the proposed CPI framework, two hypothetical future scenarios (S3 and S4) were developed where S3 included fish and livestock drinking and S4 further took in recreation use of treated effluent. Overall, both the GWQI and CWQI showed a continuous performance decline. The results revealed that CWQI presents more strict results (lower index values) for the facilities with parameters’ concentrations exceeding the target values with larger margins. As amplitude in the CWQI formulation effectively takes up this aspect, CWQI is a suitable performance measure for CPI.

The proposed CPI framework provides a platform to initiate the performance benchmarking process for WWT facilities at the local or regional levels in Saudi Arabia and elsewhere. Decision makers can include other operational, financial, and environmental indicators for the long-term sustainability of wastewater reuse in arid regions.

Despite reuse application benefits, treated effluents have a wide range of impacts on soil environment, plant growth, livestock, and public health. Presently, most of the treated effluent is reused for irrigation and landscaping purposes. In the absence of treated effluent irrigation infrastructure, the excess treated effluents discharged into the natural water bodies result in ponding conditions. Because of zero flows during dry weather conditions in the surface water bodies (such as the Wadi Rumah) of arid regions, the water quality of surface ponds needs to be assessed to ensure environmental protection and public health safety. Future studies can investigate other important factors for practical implementation of the framework, such as the training of both the suppliers and users for minimizing operational and political barriers and eliminating misapprehensions about wastewater reuse. Future research can apply the framework for assessing the impacts of heavy metals (through the food chain in the case of unrestricted irrigation) on human health and potential exposure from recreational activities. In municipal wastewater, the impacts of emerging contaminants, e.g., pharmaceuticals, fragrances, artificial sweeteners, pesticides, biocides, and disinfection by-products, can also be assessed using the proposed CPI framework.

## Figures and Tables

**Table 1:** Wastewater reuse scenarios for CPI.

No	Description	Potential Designated Uses			
		RI	URI	FLD	FR
S1	Existing scenario for reuse of wastewater for restricted irrigation (RI)	✓			
S2	Existing scenarios for reuse applications for unrestricted irrigation (URI)	✓	✓		
S3	Cycle 1 of continuous improvement for wastewater reuse for fishery and livestock drinking (FLD)	✓	✓	✓	
S4	Cycle 2 for continuous improvement for wastewater reuse for fishery and recreation uses (FR)	✓	✓	✓	✓

**Table 2:** Standards and guidelines for reuse of treated wastewater.

No.	Water Quality Parameter	Units	KSA Standards for RI ^a^	KSA Standards for URI ^a^	Recommended Standards for CPI	
			S1—Existing Situation	S2—Existing Situation	S3—CPI Cycle 1	S4—CPI Cycle 2
1.	Physical					
1.1	Total dissolved solids (TDS)	mg/L	2500	2000	1500 ^b^	<450 ^c^
1.2	Total suspended solids (TSS)	mg/L	40	10	5 ^d^	5
2.	Chemical					
2.1	pH	-	6–8.4	6–8.4	6.5–8.5 ^e^	6.5–8.0 ^c^
2.2	Biochemical oxygen demand (BOD_5_)	mg/L	40	10	5 ^f^	3 ^g^
2.3	Chemical oxygen demand (COD)	mg/L	50	50	40 ^h^	25 ^g^
2.4	Ammonia nitrogen (NH_3_–N)	mg/L	5	5	0.9 ^g^	0.3 ^g^
2.5	Nitrate nitrogen (NO_3_–N)	mg/L	10	10	10	7 ^g^
2.6	Phosphates (PO_4_–P)	mg/L	10	10 ^i^	2 ^j^	2
2.7	Residual chlorine (Cl_2_)	mg/L	0.2–0.5	0.2–0.5	0.2–0.5 ^a^	0.2–0.5 ^a^
3.	Biological					
3.1	Total coliforms (TC)	MPN/100mL	1000	10	2.2 ^k^	2.2
3.2	Fecal coliforms (FC)	MPN/100mL	1000	2.2	0 ^l^	0

^a^ KSA standards for reuse in restricted irrigation (RI) and unrestricted irrigation (URI) [38]. ^b^ Kuwait [39]; ^c^ FAO (FAO, n.d.) [40]; ^d^ New Jersey [41]; ^e^ Maryland, Massachusetts, Cyprus [40,41,42,43]; ^f^ ISO [12]; ^g^ Malaysia [44]; ^h^ China for raw vegetables [12]; ^i^ Cyprus [45]; ^j^ Italy [11]; ^k^ Food crops standards in California (U.S.A.) [46]; ^l^ Food crop standards in Massachusetts (U.S.A.), U.S. EPA [42,43].

**Table 3:** The 10-point Likert scale used to score the importance of WQPs and to define priori.

Subjective Rating	*Ŵ_j_*
Very unimportant	1
Unimportant	3
Average important	5
Important	7
Very important	9

**Table 4:** Categorization of the CCME-WQI as defined by CCME [16].

CWQI	Performance Category	Description
95–100	Excellent	Water quality is protected with a virtual absence of impairment; conditions are very close to pristine levels. These index values can only be obtained if all measurements meet recommended guidelines virtually all of the time.
89–94	Very Good	Water quality is protected with a slight presence of impairment; conditions are close to pristine levels.
80–88	Good	Water quality is protected with only a minor degree of impairment; conditions rarely depart from desirable levels.
65–79	Fair	Water quality is usually protected but occasionally impaired; conditions sometimes depart from desirable levels.
45–64	Marginal	Water quality is frequently impaired; conditions often depart from desirable levels.
0–44	Poor	Water quality is almost always impaired; conditions usually depart from desirable levels.

**Table 5:** Weighting scheme for CWQI.

No.	Importance Weight for *CWQI_PC_* (*W*_1_)	Importance Weight for *WQI_MB_* (*W*_2_)
Scenario 1	0.9	0.1
Scenario 2	0.8	0.2
Scenario 3	0.7	0.3
Scenario 4	0.7	0.3

**Table 6:** Summary of water quality monitoring data for the four WWT facilities.

Water Quality Parameters (WQPs)	Units	MIN	MEAN	MAX	SD	CV
**WWTP1**						
Total dissolved solids (TDS)	mg/L	1078	1469	3263	152	10.3
Total suspended solids (TSS)	mg/L	1.0	1.0	2.0	0.1	9.9
pH	-	6.9	7.3	8.4	0.2	2.9
Biochemical oxygen demand (BOD_5_)	mg/L	1.0	2.7	7.7	0.9	32.1
Chemical oxygen demand (COD)	mg/L	5.4	9.3	20.0	2.7	28.8
Ammonia nitrogen (NH_3_-N)	mg/L	0.0	0.5	6.4	1.2	248.8
Nitrate nitrogen (NO_3_–N)	mg/L	0.4	2.3	4.9	0.9	38.3
Phosphates (PO_4_^3-^)	mg/L	1.2	5.4	8.2	1.2	22.6
Residual chlorine (Cl_2_)	mg/L	0.3	0.7	3.8	0.3	34.4
Total coliforms (TC)	MPN/100 mL	<1	<1	<1	<1	-
Fecal coliforms (FC)	MPN/100 mL	<1	<1	<1	<1	-
**WWTP2**						
Total dissolved solids (TDS)	mg/L	673	776	1987	101	13
Total suspended solids (TSS)	mg/L	1.0	1.0	1.0	0.2	20.4
pH	-	6.7	7.2	8.3	0.3	3.6
Biochemical oxygen demand (BOD_5_)	mg/L	0.7	2.8	6.3	1.2	42.6
Chemical oxygen demand (COD)	mg/L	8.0	10.9	17.0	1.8	16.6
Ammonia nitrogen (NH_3_–N)	mg/L	0	0	0	0	-
Nitrate nitrogen (NO_3_–N)	mg/L	6.3	8.5	9.6	0.5	6.1
Phosphates (PO_4_^3-^)	mg/L	3.0	4.4	11.0	1.4	31.5
Residual chlorine (Cl_2_)	mg/L	0.5	0.9	3.9	0.3	28.1
Total coliforms (TC)	MPN/100 mL	<1	<1	<1	<1	-
Fecal coliforms (FC)	MPN/100 mL	<1	<1	<1	<1	-
**WWTP3**						
Total dissolved solids (TDS)	mg/L	877	936	1759	75	8.0
Total suspended solids (TSS)	mg/L	1.0	1.3	7.0	0.7	51.7
pH	-	7.0	7.2	8.2	0.2	2.5
Biochemical oxygen demand (BOD_5_)	mg/L	0.5	3.7	8.1	1.9	52.5
Chemical oxygen demand (COD)	mg/L	4.0	17.7	39.0	5.9	33.4
Ammonia nitrogen (NH_3_–N)	mg/L	0.0	0.2	5.0	0.8	357.6
Nitrate nitrogen (NO_3_–N)	mg/L	1.1	3.7	4.7	0.6	15.8
Phosphates (PO_4_^3-^)	mg/L	1.8	4.6	11.4	1.2	27.0
Residual chlorine (Cl_2_)	mg/L	0.5	0.9	4.0	0.3	34.3
Total coliforms (TC)	MPN/100 mL	<1	<1	<1	<1	-
Fecal coliforms (FC)	MPN/100 mL	<1	<1	<1	<1	-
**WWTP4**						
Total dissolved solids (TDS)	mg/L	823	872	1152	43.7	5.0
Total suspended solids (TSS)	mg/L	1.0	2.1	6.0	0.5	23.2
pH	-	6.5	7.0	7.6	0.1	1.7
Biochemical oxygen demand (BOD_5_)	mg/L	2.8	5.0	6.6	0.6	12.7
Chemical oxygen demand (COD)	mg/L	14.0	18.5	33.0	1.8	9.6
Ammonia nitrogen (NH_3_–N)	mg/L	0.0	0.1	0.8	0.1	46.4
Nitrate nitrogen (NO_3_–N)	mg/L	6.0	6.3	8.0	0.5	8.0
Phosphates (PO_4_^3-^)	mg/L	6.0	7.5	12.5	1.0	13.2
Residual chlorine (Cl_2_)	mg/L	0.5	0.7	2.6	0.2	29.7
Total coliforms (TC)	MPN/100 mL	<1	<1	<1	<1	-
Fecal coliforms (FC)	MPN/100 mL	<1	<1	<1	<1	-

**Table 7:** Probabilities of meeting the desired effluent standards of the WWT facilities.

Scenario	pH	TDS	TSS	BOD_5_	COD	NH_3_–N	NO_3_–N	PO_4_^3-^	Cl_2_	TC	FC
**S1:** Existing scenario for reuse of wastewater for restricted irrigation (RI)											
WWTP-1	99.75	99.50	99.75	99.75	99.75	98.24	99.75	99.75	0.50	99.99	99.99
WWTP-2	99.81	99.81	99.81	99.39	99.81	99.81	99.81	99.23	0.01	99.99	99.99
WWTP-3	99.73	99.73	99.73	98.89	99.16	99.73	99.73	99.45	0.01	99.99	99.99
WWTP-4	99.73	99.73	99.73	99.73	99.73	99.73	99.73	96.72	15.03	99.99	99.99
**S2:** Existing scenarios for reuse applications for unrestricted irrigation (URI)											
WWTP-1	99.75	98.99	99.75	99.75	99.75	98.24	99.75	99.75	0.50	99.99	99.99
WWTP-2	99.81	99.81	99.81	99.39	99.81	99.81	99.81	99.23	0.01	99.99	99.99
WWTP-3	99.73	99.73	99.73	98.89	99.16	99.73	99.73	99.45	0.01	99.99	99.99
WWTP-4	99.73	99.73	99.73	99.73	99.73	99.73	99.73	96.72	15.03	99.99	99.99
**S3:** Cycle 1 of continuous improvement for wastewater reuse for livestock drinking (LSD)											
WWTP-1	99.75	74.06	99.75	97.98	99.75	85.14	99.75	1.76	0.50	99.99	99.99
WWTP-2	99.81	99.61	99.81	94.51	99.81	99.81	99.81	0.01	0.01	99.99	99.99
WWTP-3	99.73	99.45	99.45	73.33	99.16	93.44	99.73	6.56	0.01	99.99	99.99
WWTP-4	99.73	99.73	99.45	69.67	99.73	99.45	99.73	0.01	15.03	99.99	99.99
**S4:** Cycle 2 for continuous improvement for wastewater reuse for recreation uses (RC)											
WWTP-1	97.98	0.005	99.75	72.29	99.75	83.63	99.75	1.76	0.50	99.99	99.99
WWTP-2	98.84	0.008	99.81	60.37	99.81	99.81	0.19	0.01	0.01	99.99	99.99
WWTP-3	98.91	0.009	99.45	37.78	88.24	92.62	99.73	6.56	0.01	99.99	99.99
WWTP-4	99.73	0.010	99.45	3.01	99.45	99.45	98.09	0.01	15.03	99.99	99.99

**Table 8:** Importance weights estimated using entropy method for all the scenarios.

Scenario	WQPs											Sum
	pH	TDS	TSS	BOD5	COD	NH_3_-N	NO_3_-N	PO_4_	Cl_2_	TC	FC	
S1	0.05	0.11	0.11	0.09	0.09	0.09	0.17	0.17	0.03	0.04	0.04	1
S2	0.06	0.09	0.08	0.07	0.07	0.07	0.13	0.13	0.07	0.10	0.10	1
S3	0.06	0.09	0.07	0.07	0.07	0.07	0.11	0.11	0.10	0.12	0.12	1
S4	0.07	0.07	0.08	0.07	0.07	0.07	0.11	0.11	0.10	0.12	0.12	1

**Table 9:** Proposed improvement actions for continuous performance improvement (CPI) of WWTPs.

CPI ^a^ Scenario	Proposed Improvement Actions for CPI
S1: RI ^b^	Careful monitoring and process control. Attention is required for controlling residual chlorine.
S2: RI + URI ^c^	Careful monitoring and process control. Attention is required for controlling residual chlorine.
S3: RI + URI + FLD ^d^	Careful monitoring and process control. Improve nutrients removal by integrating the existing system with membrane bioreactor (MBR) process.
S4: RI +URI + FLD + FR ^e^	Careful monitoring and process control. Improve nutrients removal by integrating the existing system with membrane bioreactor (MBR) process. Upgrade tertiary treatment by adding reverse osmosis process for the removal of total dissolved solids (TDS) and dissolved organics.

^a^ Continuous performance improvement (CPI); ^b^ Restricted irrigation (RI); ^c^ Unrestricted irrigation (URI); ^d^ fishery and livestock drinking (FLD); ^e^ Fishery and recreation (FR)

## Data Availability

Detailed data cannot be shared due to the confidentiality contract between the data sharing and research organizations.

## Appendix A. Application of Entropy Method

Table A1 presents the decision matrix for scenario 1 about the four criteria and eleven water quality parameters.

**Table A1:** Decision matrix for scenario 1 (restricted irrigation).

No.	Evaluation Criteria	Water Quality Parameters										
		pH	TDS	TSS	BOD5	COD	NH_3_–N	NO_3_–N	PO_4_	Cl_2_	TC	FC
C1	Impact on geoenvironment	5	7	9	7	7	7	9	9	3	5	5
C2	Impact on plant growth	7	9	7	7	7	7	9	9	5	5	5
C3	Impact on livestock drinking	1	1	1	1	1	1	1	1	1	1	1
C4	Impact on possible human contact	1	1	1	1	1	1	1	1	1	1	1

Table A2 presents the entropy matrix (*E_j_*) developed using Equation (3).

**Table A2:** Entropy matrix.

E1	E2	E3	E4	E5	E6	E7	E8	E9	E10	E11
−15.63	−24.09	−24.09	−19.65	−19.65	−19.65	−28.53	−28.53	−8.18	−11.61	−11.61

Table A3 shows importance weights (*w_j_*) estimated using Equation (5), degree of diversity (*d_j_*) using Equation (6) priori using Table 3 of the main text, and the final weights (*W_j_*) estimated using Equation (7).

**Table A3:** Weights estimation matrix.

Water Quality Parameters	pH	TDS	TSS	BOD5	COD	NH_3_-N	NO_3_-N	PO_4_	Cl_2_	TC	FC
Importance weights (*w_j_*)	0.0748	0.1129	0.1129	0.0929	0.0929	0.0929	0.1329	0.1329	0.0413	0.0567	0.0567
Priori ( ${\hat{w}}_{j}$ )	5	7	7	7	7	7	9	9	5	5	5
Weights (*Wj*)	0.05	0.11	0.11	0.09	0.09	0.09	0.17	0.17	0.03	0.04	0.04
